# IL-4 Deficiency Is Associated with Mechanical Hypersensitivity in Mice

**DOI:** 10.1371/journal.pone.0028205

**Published:** 2011-12-02

**Authors:** Nurcan Üçeyler, Tengü Topuzoğlu, Peter Schießer, Saskia Hahnenkamp, Claudia Sommer

**Affiliations:** Department of Neurology, University of Würzburg, Würzburg, Germany; University of Muenster, Germany

## Abstract

Interleukin-4 (IL-4) is an anti-inflammatory and analgesic cytokine that induces opioid receptor transcription. We investigated IL-4 knockout (ko) mice to characterize their pain behavior before and after chronic constriction injury (CCI) of the sciatic nerve as a model for neuropathic pain. We investigated opioid responsivity and measured cytokine and opioid receptor gene expression in the peripheral and central nervous system (PNS, CNS) of IL-4 ko mice in comparison with wildtype (wt) mice. Naïve IL-4 ko mice displayed tactile allodynia (wt: 0.45 g; ko: 0.18 g; p<0.001), while responses to heat and cold stimuli and to muscle pressure were not different. No compensatory changes in the gene expression of tumor necrosis factor-alpha (TNF), IL-1β, IL-10, and IL-13 were found in the PNS and CNS of naïve IL-4 ko mice. However, IL-1β gene expression was stronger in the sciatic nerve of IL-4 ko mice (p<0.001) 28 days after CCI and only IL-4 ko mice had elevated IL-10 gene expression (p = 0.014). Remarkably, CCI induced TNF (p<0.01), IL-1β (p<0.05), IL-10 (p<0.05), and IL-13 (p<0.001) gene expression exclusively in the ipsilateral spinal cord of IL-4 ko mice. The compensatory overexpression of the anti-inflammatory and analgesic cytokines IL-10 and IL-13 in the spinal cord of IL-4 ko mice may explain the lack of genotype differences for pain behavior after CCI. Additionally, CCI induced gene expression of μ, κ, and δ opioid receptors in the contralateral cortex and thalamus of IL-4 ko mice, paralleled by fast onset of morphine analgesia, but not in wt mice. We conclude that a lack of IL-4 leads to mechanical sensitivity; the compensatory hyperexpression of analgesic cytokines and opioid receptors after CCI, in turn, protects IL-4 ko mice from enhanced pain behavior after nerve lesion.

## Introduction

The immune system plays a major role in the induction and maintenance of neuropathic pain [1], [2]. Balanced interactions between immune cells, immune cell modulatory molecules, cytokines, and chemokines are necessary for pain homeostasis. The major implication of this balance becomes apparent when parts of the network are missing or dysfunctional. For instance, lack of the T-cell co-inhibitory molecule B7-H1 leads to increased T-cell proliferation and pro-inflammatory cytokine production with prolonged pain behavior after peripheral nerve lesion [3]. In many animal models, pro-inflammatory cytokines are algesic and anti-inflammatory cytokines are analgesic, see [4] for review. The anti-inflammatory cytokines interleukin (IL)-4, IL-10, and IL-13 have analgesic effects in inflammatory and neuropathic pain models [5]–[10]. IL-4 and IL-13 share the same receptor and act via the signal transducer and activator of transcription 6 (STAT6) pathway. IL-4 is of particular interest, since it links the immune system to the opioid system by inducing μ and δ opioid receptor (MOR, DOR) transcription [11], [12].

In humans an imbalance of the expression of pro- and anti-inflammatory cytokines is assumed to be one factor in inducing and maintaining pain. We recently showed altered local and systemic cytokine profiles in patients with several different chronic pain disorders [13]–[16]. Reduced systemic expression of IL-4 and IL-10 was found in most of these patients. We therefore hypothesized that this reduction of anti-inflammatory cytokines might be causally related to pain and that IL-4 knockout (ko) mice would display altered pain behavior. Since IL-4 induces MOR and DOR transcription in neuronal cell cultures [11], [12], we further hypothesized that IL-4 ko mice might respond differently to morphine treatment. We used chronic constriction injury (CCI) of the right sciatic nerve as a model of neuropathic pain and set out to characterize IL-4 ko mice as to their pain behavior, opioid response and cytokine and opioid receptor gene expression before and after CCI. We show that IL-4 deficiency leads to mechanical allodynia and changes spinal cytokine regulation after CCI.

## Methods

### Ethic statement

All experiments were approved by the Bavarian State authorities (Regierung von Unterfranken, # 02/08). Mice were held at the animal facilities of the Department of Neurology, University of Würzburg under standard conditions with food and water access ad libitum. Animal use and care were in accordance with the institutional guidelines.

### Animals and surgery

We investigated 30 IL-4 ko mice and 30 wild type (wt) mice of C57Bl/6J background. The mean age was eight to ten weeks. IL-4 ko breeder pairs on C57Bl/6J background were purchased from Jackson Laboratories (Maine, USA), wt C57Bl/6J animals were purchased from Charles River Laboratories (Sulzfeld, Germany).

Ten mice of each group received CCI at the right sciatic nerve, while ten mice served as unoperated controls. Further 10 mice per genotype were used for the pharmacological experiments (see below). The procedure of CCI of the sciatic nerve followed the original description [17] with modifications for mice [18]. In brief, the right sciatic nerve was exposed under intraperitoneal (i.p.) isoflurane narcosis and three ligatures (7-0 prolene) were placed around the sciatic nerve proximal to the trifurcation with a distance of one mm between each ligature. The ligatures were loosely tied until a short flick of the ipsilateral hind limb was observed.

### Behavioral tests

All behavioral tests were performed by an experienced investigator blinded as to genotype and intervention. Paw *withdrawal latencies to heat* were measured according to the method of Hargreaves [19] applying a standard Ugo Basile Algesiometer (Comerio, Italy). The animals were placed on a glass surface and a radiant heat source was positioned under one hind paw. The time to paw withdrawal was recorded automatically. To avoid tissue damage the time limit for heat application was set to 15 seconds. Each hind paw was tested three times.

The von-Frey test based on the up-and-down-method was used to test for the paw *withdrawal thresholds to tactile stimulation* [20]. Animals were placed in plexiglass cages on a wire mesh. The plantar surface of the hind paws was touched with a von-Frey monofilament starting at a hair value of 0.69 g. When the animal withdrew its hind paw upon administration of mild pressure the next finer von-Frey filament was used. If the animal did not react to this stimulation, the next stronger von-Frey filament was applied. Each hind paw was tested three times. The 50% withdrawal threshold (i.e. force of the von-Frey hair to which an animal reacts in 50% of the administrations) was recorded.

The acetone test was used to investigate paw *withdrawal latencies to cold*. Mice were placed in plexiglass cages on a wire mesh. The plantar surface of the hind paw was touched with a drop of water at body temperature as a control condition and the reaction of the mouse was recoded (paw withdrawal or no paw withdrawal). Afterwards the paw was dried and a drop of acetone was applied. The total time of paw withdrawal (i.e. time until the withdrawn hind paw was placed back on the wire mesh for at least two seconds) was recorded. The cut off time was 30 seconds.

Using the Randall-Selitto test (Ugo Basile algesimeter, Chicago, USA) the *withdrawal latency to muscle pressure* was investigated. The test was applied to test for muscle tenderness, which is a leading clinical feature in patients with chronic pain and reduced IL-4 levels [13]. The animal was held with the head in a cotton sock for calming and the gastrocnemius muscle was placed in the Randall-Selitto device. Pressure to the muscle was slowly increased until the animal withdrew its hindlimb. The time until hindlimb withdrawal was recorded.

The *withdrawal latencies to heat* and the *withdrawal thresholds to von-Frey hairs* were performed at baseline and up to day 28 after CCI at regular time points (twice prior to CCI; days 3, 7, 9, 14, and 28 after CCI). The *acetone test* and the test for *withdrawal latencies to muscle pressure* were performed in naïve mice of both genotypes at baseline only to keep the experimental stress on the animals limited.

### Pharmacological treatment

At day seven after CCI five mice of each group received morphine i.p. (Merck, Germany) at a dosage of 10 mg/kg body weight. The choice of this dose based on pilot studies showing the best analgesic effect with least behavioral disturbance. Another five mice in each group received normal saline as a control. Behavioral tests were performed at 2, 4, and 6 hours after morphine administration.

### Tissue collection

Twenty-eight days after CCI mice were exsanguinated and euthanized in deep isoflurane anesthesia and tissue was collected for qRT-PCR studies. At this time point pain behavior was still present in CCI mice of both genotypes. The ipsi- and contralateral sciatic nerves and the ipsi- and contralateral lumbar dorsal root ganglia (DRG) L4 and L5 were dissected. The collected ipsilateral nerve contained the segment from under the ligatures to 1 cm proximal to the first ligature. An equivalent segment of the contralateral sciatic nerve was collected. Additionally, the frontal cortex, hippocampus, hypothalamus, thalamus, pons, and the lumbar and cervical spinal cord were dissected. Except for the control animals, tissue from the ipsi- and contralateral side was collected separately. For the correct localisation of the dissected brain areas we used the online mouse brain library (www.mbl.org). Tissue was dissected for qRT-PCR using a dissection microscope (Zeiss, Oberkochen, Germany) and was immediately flash-frozen in liquid nitrogen and stored at −80°C before further processing.

### Gene expression analysis

*mRNA extraction:* mRNA extraction from mouse nervous tissue was performed following the method of Chomczynski [21] with little modification as described elsewhere [22].

*Reverse transcription PCR:* All PCR reagents and cyclers were obtained from Applied Biosystems, Darmstadt, Germany. For reverse transcription PCR we used TaqMan Reverse Transcription Reagents®. The 100 µl reaction contained 500 ng of mRNA and the following reagents (per sample): 10× Reaction Buffer (10 µl), 10 mM dNTPs (20 µl), 25 mM MgCl_2_ (22 µl), Random Hexameres (5 µl), RNAse Inhibitor (2 µl) and 50 U/µl Multiscribe Reverse Transcriptase (6.25 µl). The 96-well GeneAmp® PCR System 9700 cycler was employed for the reaction. The cycler conditions were: 10 min, 38°C; 60 min, 48°C; 25 min, 95°C.

*qRT-PCR:* The Assay-IDs of the investigated target genes are given in brackets. We investigated the relative gene expression of the pro-inflammatory cytokines tumor necrosis factor-alpha (TNF; Mm00443258_m1), IL-1β (Mm00434228_m1), the anti-inflammatory cytokines IL-10 (Mm00439616_m1), and IL-13 (Mm00434204_m1) in the peripheral nervous system and the spinal cord. IL-13 was chosen because it shares the IL-4 receptor and several IL-4 functions and may be compensatorily regulated.

Gene expression of the opioid receptors μ (Mm00440568_m1), δ (Mm00443063_m1), and κ (Mm00440561_m1) were investigated in the brain (frontal cortex, hippocampus, hypothalamus, thalamus, and pons) and in the ipsi- and contralateral spinal cord. Five µl of cDNA were taken for qRT-PCR, which was performed in the GeneAmp 7700 sequence detection system® capable of fluorescence measuring using TaqMan Universal Master Mix® as previously described [22].

### Statistical analysis

For statistical analysis and graph design SPSS software Version 17 was employed (Munich, Germany). The data of behavioral testing showed normal distribution in the Kolmogorow-Smirnow-test. Therefore ANOVA with post hoc Tukey correction was used for group comparisons. The results of the behavioral tests are illustrated as dot plots giving the mean and the standard error of the mean (SEM). Since our mRNA data did not show a normal distribution we used the non-parametric Mann-Whitney-U-test for pairwise comparison of the distinct time points with Bonferroni correction. Statistical significance was assumed at p<0.05. The qRT-PCR data are illustrated as box- and whisker plots giving the median, the upper 75% and lower 25% percentiles and the minimum and maximum values.

## Results

### IL-4 ko mice are hypersensitive to tactile stimuli

**Figure 1:**
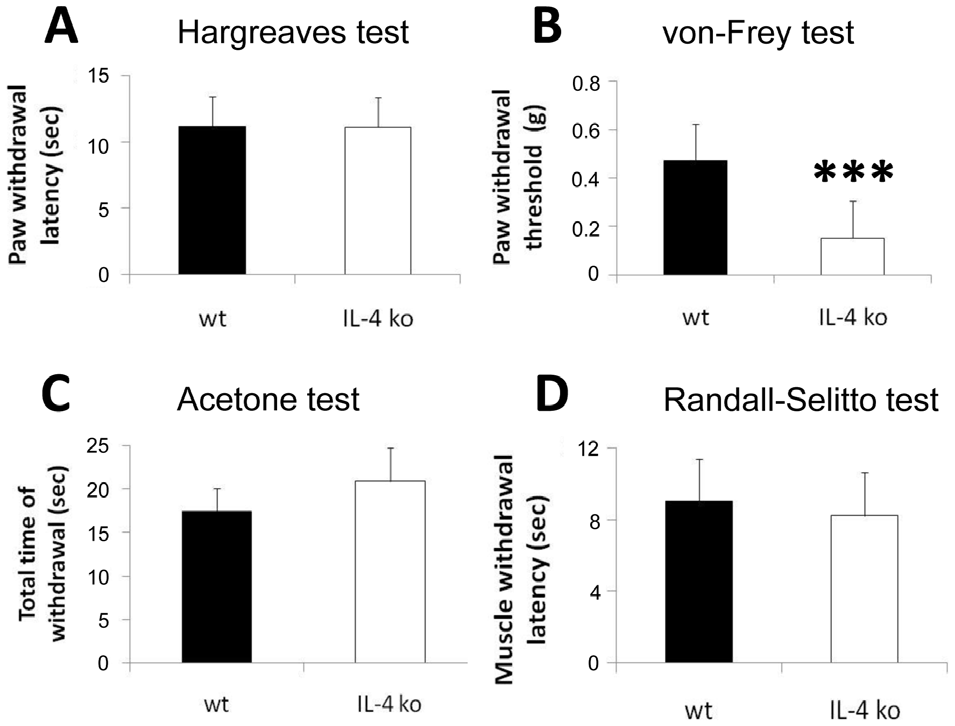
Behavioral tests in naïve IL-4 ko and wt mice. The bars illustrate the results of the behavioral tests in naïve wt and IL-4 ko mice. A) IL-4 ko mice do not differ from wt mice in withdrawal latencies to heat. B) IL-4 ko mice have reduced paw withdrawal thresholds to mechanical stimulation with von-Frey filaments (***p<0.001). IL-4 ko mice do not differ from wt mice in paw withdrawal time to acetone (C), and in withdrawal latencies of the hind limb upon pressure to the gastrocnemius muscle (D).

While baseline paw withdrawal latencies to heat were not different between naïve IL-4 ko and wt mice (Fig. 1A), naïve IL-4 ko mice had lower paw withdrawal thresholds to von-Frey hairs indicative of mechanical allodynia (p<0.001; Fig. 1B). Paw withdrawal latencies upon cold stimulation and hind limb withdrawal latencies upon muscle pressure were not different between genotypes (Fig. 1C, D).

**Figure 2:**
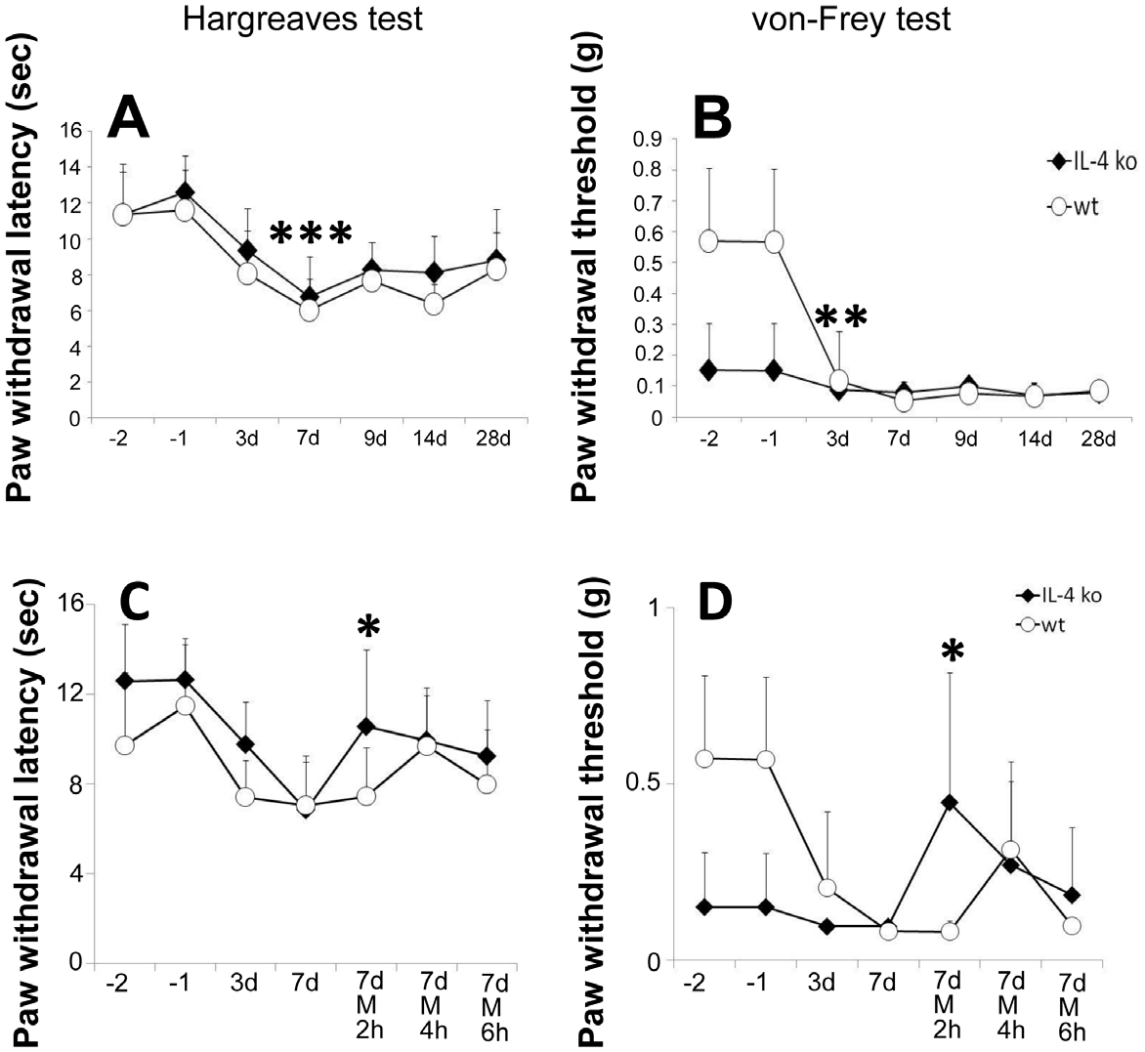
Analgesic effect of morphine in IL-4 ko and wt mice. Paw withdrawal latencies to thermal stimuli and withdrawal thresholds to von Frey hairs in wt and IL-4 ko mice after CCI (A, B) and additional morphine treatment (C, D). CCI leads to thermal (A) and mechanical (B) hypersensitivity lasting up to day 28 after CCI. Mice received morphine i.p. (abbreviated as M in the graphs) at day seven post-surgery. Only IL-4 ko mice showed elevation of mechanical withdrawal thresholds and prolongation thermal withdrawal latencies at 2 h after morphine i.p. (A, B). Asterisk: *p<0.05, **p<0.01, ***p<0.001.

After CCI both IL-4 ko and wt mice developed thermal hypersensitivity (p<0.001 wt and ko; Fig. 2A) and wt mice developed mechanical hypersensitivity (wt: p = 0.009; Fig. 2B), which started three days after injury and lasted up to 28 days, without differences between genotypes. IL-4 ko mice did not display a further decrease in mechanical withdrawal thresholds (Fig. 2B). Unexpectedly, only IL-4 ko mice showed an analgesic response in the tests for thermal (p = 0.02; Fig. 2C) and mechanical sensitivity (p = 0.046; Fig. 2D) two hours after morphine administration at day seven post surgery, while withdrawal latencies and thresholds did not normalize in wt mice.

### CCI induces a stronger pro- and anti-inflammatory cytokine response in the sciatic nerve of IL-4 ko mice and increases cytokine gene expression in the ipsilateral spinal cord exclusively in IL-4 ko mice

Naïve IL-4 ko and wt mice did not differ in their TNF, IL-1β, IL-10, and IL-13 gene expression in sciatic nerve, lumbar and cervical spinal cord, and DRG. IL-13 was undetectable in sciatic nerve of both genotypes, as previously described [23].

**Figure 3:**
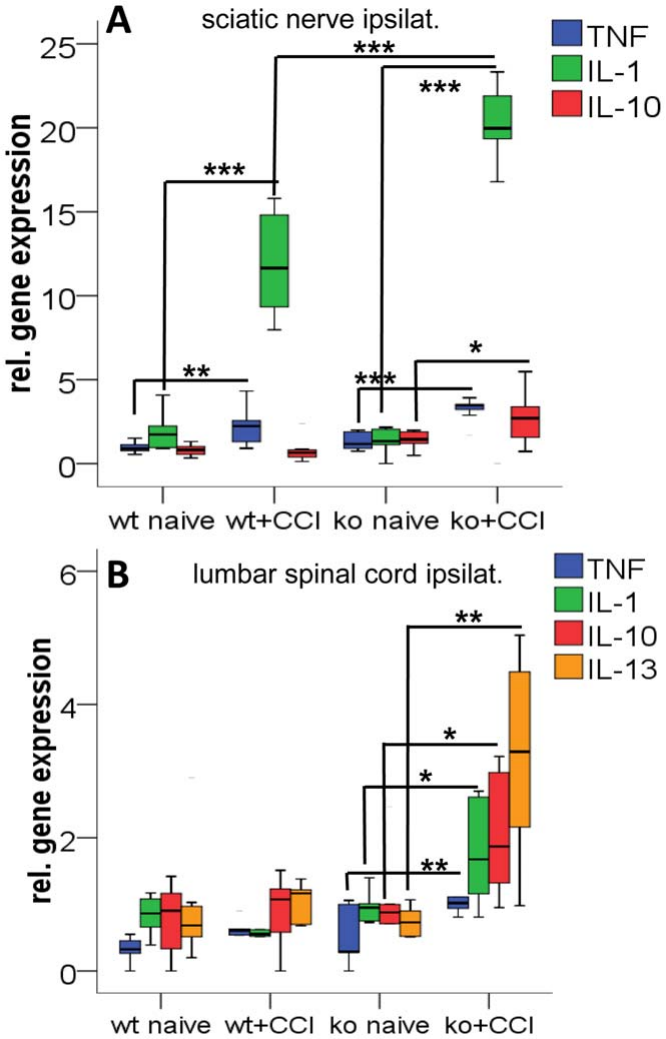
Cytokine gene expression before and after CCI. The box- and whisker plots illustrate the relative gene expression of pro- and anti-inflammatory cytokines in the ipsilateral sciatic nerve (A), and ipsilateral lumbar spinal cord (B) 28 days after CCI. A) CCI leads to an increase in TNF and IL-1ß gene expression in the ipsilateral sciatic nerve. The increase in IL-1ß gene expression is higher in IL-4 ko mice compared to wt mice. IL-10 gene expression increases only in IL-4 ko mice. B) CCI leads to an increase in TNF, IL-1ß, IL-10, and IL-13 gene expression only in IL-4 ko mice; this increase is higher compared to wt mice after CCI (TNF: p = 0.002; IL-1ß: p<0.001; IL-10: p = 0.011; IL-13: p = 0.026). Asterisk: *p<0.05, **p<0.01, ***p<0.001.

Four weeks after CCI, both IL-4 ko and wt mice displayed elevated TNF (ko: p<0.001; wt: p = 0.007) and IL-1β (p<0.001 ko and wt; Fig. 3A) gene expression in the ipsilateral sciatic nerve compared to unoperated control animals. The increase in IL-1β gene expression was higher in IL-4 ko mice (p<0.001) and only IL-4 ko mice had elevated IL-10 gene expression (p = 0.014; Fig. 3A). In the lumbar spinal cord only IL-4 ko mice displayed elevated TNF (p = 0.001), IL-1β (p = 0.024), IL-10 (p = 0.032), and IL-13 (p = 0.001; Fig. 3B) gene expression four weeks after CCI. This increase was higher compared to wt mice after CCI (TNF: p = 0.002; IL-1ß: p<0.001; IL-10: p = 0.011; IL-13: p = 0.026).

### CCI induces an increase in opioid gene expression in the contralateral thalamus of IL-4 mice

**Figure 4:**
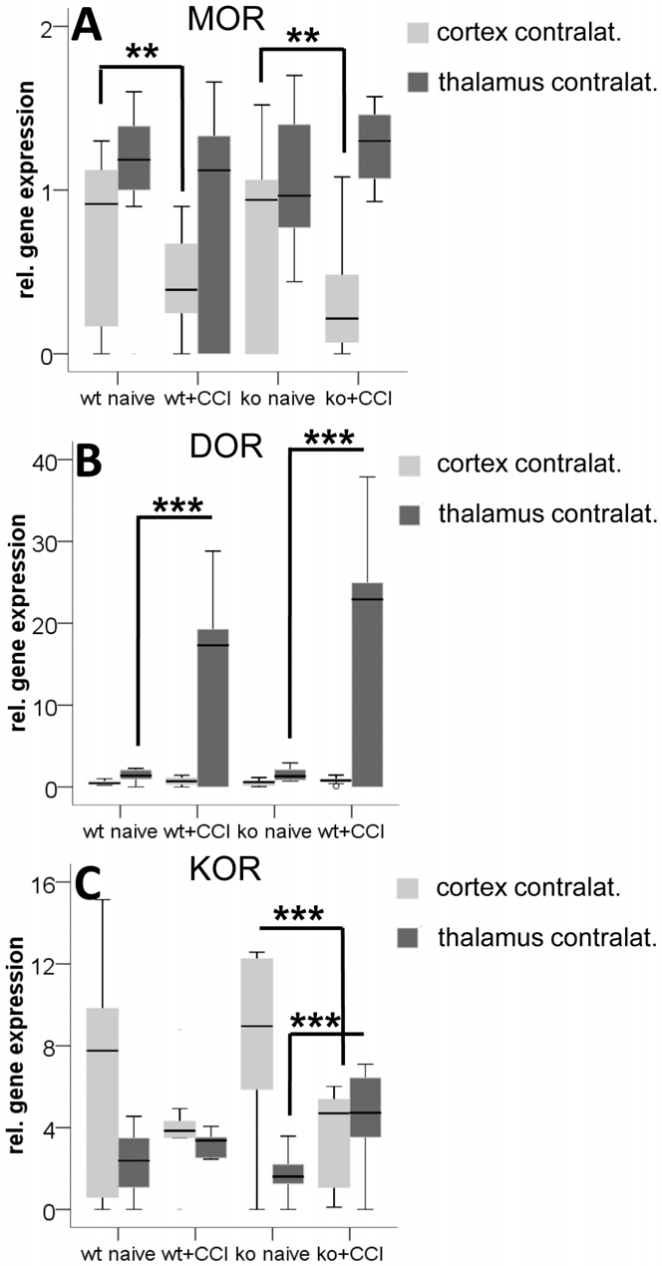
Cerebral opioid receptor gene expression in IL-4 ko and wt mice before and after CCI. The box- and whisker plots illustrate the relative gene expression of the opioid receptors MOR (A), DOR (B), and KOR (C) in the contralateral frontal cortex and thalamus at day 28 after CCI. A) MOR gene expression decreases in the contralateral frontal cortex in IL-4 ko mice and in wt mice. B) DOR gene expression increases in the contralateral thalamus in both wt and IL-4 ko mice. C) KOR gene expression decreases in the contralateral frontal cortex of IL-4 ko and wt (n.s.) mice and increases in the contralateral thalamus only in IL-4 ko mice. Asterisk: *p<0.05, **p<0.01, ***p<0.001.

**Figure 5:**
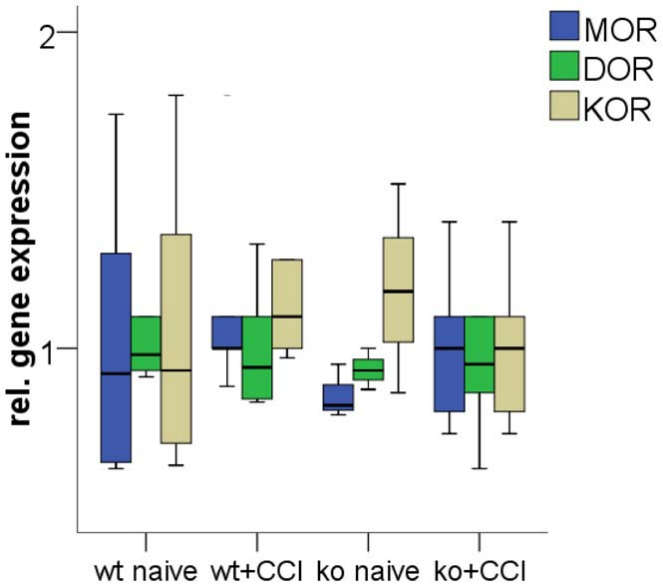
Spinal opioid receptor gene expression in IL-4 ko and wt mice before and after CCI. The box- and whisker plots illustrate the relative gene expression of the opioid receptors MOR, DOR, and KOR in the lumbar spinal cord of naïve wt and IL-4 ko mice and at day 28 after CCI (data shown for contralateral spinal cord sections; also no changes in opioid receptor gene expression were found in the inpsilateral spinal cord (data not shown). No intergroup difference is found before and after nerve lesion.

Baseline gene expression of MOR, DOR, and KOR in the investigated brain areas frontal cortex, thalamus, hypothalamus, and pons of naïve mice did not differ between genotypes. At 28 days after CCI, gene expression of the three opioid receptors did not change in pons and hypothalamus of both genotypes, which served as control areas. In the *contralateral frontal cortex* MOR gene expression decreased after CCI in IL-4 ko mice and in wt mice (ko: p = 0.003; wt: p = 0.004; Fig. 4A). DOR gene expression did not change in both genotypes (Fig. 4B). KOR gene expression decreased both in wt mice (n.s.) and in IL-4 ko mice (p<0.001; Fig. 4C). In the *contralateral thalamus* MOR did not change in wt mice and increased tendentially in IL-4 ko mice (n.s.; Fig. 4A). DOR gene expression increased in both genotypes (p<0.001, Fig. 4B) and was higher in IL-4 ko mice (n.s.). KOR gene expression increased only in IL-4 ko mice (p<0.001; Fig. 4C). After CCI only KOR gene expression in the contralateral thalamus was higher in IL-4 ko mice compared to wt mice (p = 0.056). In the spinal cord we found no intergroup difference for the gene expression of MOR, KOR, and DOR and CCI did not lead to changes in the expression levels of these genes in wt and IL-4 ko mice (Fig. 5).

## Discussion

The anti-inflammatory cytokine IL-4 has analgesic effects in animal models [5], [7] and there is evidence from clinical studies that low IL-4 levels may be associated with pain [13], [14]. We used IL-4 deficient mice to investigate whether an innate lack of IL-4 leads to increased pain behavior and altered morphine responsiveness compared to wt mice, since IL-4 is one important inducer of opioid receptor transcription [11], [12]. Our main findings are that naïve IL-4 ko mice indeed are hypersensitive to tactile stimuli and that CCI leads to an increase in spinal pro- and especially anti-inflammatory cytokine gene expression exclusively in IL-4 ko mice.

Studies using cytokine ko mice to investigate pain behavior have given ample evidence for the algesic effect of pro-inflammatory cytokines: for instance, mice with an innate lack of IL-1 [24] or IL-6 [25] display reduced pain behavior - either naïve or after nerve injury. The investigation of the innate lack of anti-inflammatory cytokines and systems in pain development and maintenance has given complementary results: IL-10 deficiency is associated with enhanced pain behavior [26]; the lack of the co-inhibitory molecule B7-H1 leads to mechanical hypersensitivity, increased pro-inflammatory cytokine gene expression and prolonged pain behavior after CCI [3]. Here we show that naïve IL-4 ko mice have tactile hypersensitivity. Having excluded innate differences in the expression of the other major pro- and anti-inflammatory cytokines in naïve IL-4 ko mice, we consider it very likely that the lack of IL-4 and the tactile hypersensitivity are causally related.

It is not surprising that IL-4 deficiency leads to tactile hypersensitivity and spares thermal sensitivity. Thermal and tactile sensations follow separate spinal pathways and can be affected separately. It is known that depending on modality (e.g. mechanical vs. thermal) and status (naïve vs. after intervention) pain behavior in cytokine ko mice is differentially controlled [27]–[30]. For example, lesion of dorsal column neurons can selectively attenuate tactile allodynia [28]. Why lack of IL-4 leads to mechanical allodynia in the naïve animal is not clear yet. It is well known that pro-inflammatory cytokines like IL-1 or TNF can influence electrical excitability of nociceptive neurons [31], [32]. Few studies have investigated the potential role of IL-4 in cell excitability. In an early study IL-4 was shown to activate ion channels on B-lymphocytes [33]. IL-4 also induces rapid and big increases in the activity of large-conductance, calcium-activated potassium channels in smooth muscle cells [34]. IL-4 also affects the electrical properties of spinal neurons by modifying the function of ion currents [35]. One possibility to explain mechanical allodynia in naïve IL-4 ko mice might therefore be that IL-4 ko mice have a reduction in their central inhibitory neurotransmission. In pilot experiments the analysis of spinal single unit recordings gave first indications towards an increased firing frequency of lamina V sensory neurons in IL-4 ko mice upon stimulation with an innocuous von-Frey hair (Schießer, Üçeyler, Sommer unpublished data).

Naïve IL-4 ko mice are hypersensitive to mechanical stimulation and no further drop of the mechanical withdrawal thresholds is observed after CCI. This is mostly due to the fact that the mechanical withdrawal thresholds are already very low at baseline and a further reduction exceeds the sensitivity of behavioral tests. The exclusive increase of pro- but especially anti-inflammatory cytokines in the ipsilateral spinal cord of IL-4 ko mice may also play a role. IL-4 and IL-10 expression can attenuate mechanical allodynia [36]. The lack of IL-4 alone with obviously no compensatory change in cytokine expression, as in the naïve mice, leads to the phenotype of tactile hypersensitivity. However, if the system is challenged e.g. by CCI then especially anti-inflammatory and analgesic cytokines are upregulated much stronger compared to wt mice and protect the organism from further pain.

Opioid receptor gene expression did not differ between naïve IL-4 ko and wt mice in brain and spinal cord. Interestingly, morphine responses were observed only in IL-4 ko mice after CCI. This was paralleled by an increase in contralateral thalamic DOR and KOR gene expression exclusively in IL-4 ko mice, whereas wt mice only had an increase in contralateral thalamic DOR. Although a key inducer of opioid receptors is missing (i.e. IL-4), this rapid opioid receptor gene regulation probably is due to triggers of opioid receptor expression that are more active in IL-4 ko mice: 1) Pain itself is a potent central opioid receptor inducer [25]. Taking into account that naïve IL-4 ko mice already have mechanical hypersensitivity it is plausible that a further pain trigger like CCI leads to a compensatory stronger opioid receptor gene expression; 2) Other cytokines are also potent inducers of the opioid receptor and peptide production [26], [27], [33] and IL-4 ko mice displayed a much stronger pro- and anti-inflammatory response. The finding that KOR and MOR gene expression did not change or even decreased in contralateral cortex and thalamus of wt mice after peripheral nerve lesion is in keeping with earlier studies [37]. In a recent functional study MOR-mediated G-protein activity was also altered by CCI and there was a brain region specific decrease in MOR sensitivity [38]. In a human study chronic pain caused by a peripheral nerve lesion led to a decrease of central MOR availability [39].

Of note, our gene expression analysis was performed on tissue collected at day 28 after CCI. In an earlier study we had shown that spinal gene expression of pro- and anti-inflammatory cytokines drops within the first six hours after CCI, but is back to normal values already after 10–12 hours and keeps normal up to seven days in wt mice [40]. In rat lumbar spinal cord TNF, IL-1, and IL-6 gene expression peaked between day 1 and 6 after CCI and were back to normal at day 28 [41]. Our results for absent spinal cytokine regulation in wt mice 28 days after CCI are therefore in keeping with the literature. The increase in spinal cytokine expression in IL-4 ko mice is therefore intriguing and points to a disinhibition in spinal cytokine regulation in this genotype after nerve injury.

In conclusion our study shows, that 1) naïve IL-4 ko mice are mechanically hypersensitive, while CCI does not lead to behavioral changes other than in wt mice up to 28 days after surgery; 2) morphine responsiveness occurs even earlier in IL-4 ko mice; 3) cytokine and opioid gene expression is not different between naïve genotypes; however, CCI leads to a disinhibited increase in peripheral and spinal pro- and anti-inflammatory gene expression and also to an increase in contralateral thalamic opioid receptor gene expression in IL-4 ko mice. The cytokine-pain relation obviously is more complex than a unidimensional connection between the lack of one cytokine to measurable behavioral changes. However, the fact that IL-4 deficiency results in increased pain behavior upon tactile mechanical stimulation is intriguingly similar to findings in patients with chronic widespread pain [42] [Üçeyler, Sommer unpublished data] and further studies addressing downstream mechanisms will help to better understand the role of IL-4 in pain.
